# Chronic Exposure to Two Regimens of Waterpipe Smoke Elicits Lung Injury, Genotoxicity, and Mitochondrial Impairment with the Involvement of MAPKs Activation in Mice

**DOI:** 10.3390/ijms26010430

**Published:** 2025-01-06

**Authors:** Naserddine Hamadi, Suhail Al-Salam, Sumaya Beegam, Nur Elena Zaaba, Ozaz Elzaki, Abderrahim Nemmar

**Affiliations:** 1 Department of Environmental Sciences and Sustainability, College of Natural and Health Sciences, Zayed University, Abu Dhabi P.O. Box 144534, United Arab Emirates; naserddine.hamadi@zu.ac.ae; 2 Department of Pathology, College of Medicine and Health Sciences, United Arab Emirates University, Al Ain P.O. Box 15551, United Arab Emirates; suhaila@uaeu.ac.ae; 3 Zayed Center for Health Sciences, United Arab Emirates University, Al Ain P.O. Box 15551, United Arab Emirates; 4 Department of Physiology, College of Medicine and Health Sciences, United Arab Emirates University, Al Ain P.O. Box 15551, United Arab Emirates; sumayab@uaeu.ac.ae (S.B.); elenazaaba@uaeu.ac.ae (N.E.Z.); ozazelzaki@uaeu.ac.ae (O.E.)

**Keywords:** waterpipe smoke, occasional, regular, lung, inflammation

## Abstract

While the pulmonary effects of regular waterpipe smoking (R-WPS) are well-defined, the impact of occasional waterpipe smoking (O-WPS) on the lungs remains less established. This study investigated the pulmonary toxicity and underlying mechanisms of O-WPS versus R-WPS following 6 months of exposure, focusing on histopathology, inflammation in the lung, bronchoalveolar lavage fluid (BALF), and plasma, as well as oxidative stress, genotoxicity, mitochondrial dysfunction, and the expression of mitogen-activated protein kinases (MAPKs) in lung homogenates. Exposure to both O-WPS and R-WPS resulted in significant histological changes, including increased numbers of alveolar macrophages and lymphocytes, as well as interstitial fibrosis. Only R-WPS increased the number of neutrophil polymorphs and plasma cells. R-WPS also significantly increased the chemokines CXCL1, CXCL2, and CCL2 in the lung, BALF, and plasma, while O-WPS increased CXCL1 and CXCL2 in the lung and CXCL1 in the plasma. Both exposure regimens significantly increased lung injury markers, including matrix metalloproteinase-9 and myeloperoxidase. Additionally, R-WPS induced a significant increase in the cytokines IL1β, IL6, and TNFα in the lung, BALF, and plasma, while O-WPS elevated IL1β and IL6 in the lung. Oxidative stress was observed, with increased levels of thiobarbituric acid reactive substances and superoxide dismutase in both the O-WPS and R-WPS groups. Exposure to either O-WPS or R-WPS triggered genotoxicity and altered mitochondrial complex activities. R-WPS exposure also resulted in elevated expression of p-JNK/JNK, p-ERK/ERK, and p-p38/p38, while O-WPS augmented the p-ERK/ERK ratio in the lungs. Taken together, these findings indicate that both O-WPS and R-WPS contribute to lung injury and induce inflammation, oxidative stress, genotoxicity, and mitochondrial dysfunction, with R-WPS having a more pronounced effect. These effects were associated with the activation of MAPKs.

## 1. Introduction

Waterpipe smoking (WPS) “also known as Hookah, Shisha, Arghile, and Maassal” has been in use around the world for not less than four centuries [1]. The World Health Organization asserts that WPS is a public health problem [2]. An alarming report indicated that over the past 20 years, WPS has surpassed cigarette smoking in popularity among young people [3]. Several factors may have contributed amply to the tremendous increase in WPS usage [4]. Firstly, is the belief that tobacco smoke is filtered through the water before being inhaled by smokers, which renders it less detrimental than traditional cigarettes. The latter is now being disapproved by scientific evidence [5,6,7]. Secondly, its affordability is due to heavy taxes applied on cigarettes [5,8]. Thirdly, additional positive attributes are the association of WPS practice with fun, pleasure, and relaxation [9]. Finally, there are misperceptions about the addictive potential of WPS [10]. 

It has been demonstrated that WPS has higher concentrations of toxicants than cigarette smoke (CS), which may exacerbate the harmful effects of tobacco on the lungs [11]. Bhatnagar et al. have shown an increase of nearly 8-fold in carbon monoxide after a single hookah smoking session compared with smoking one cigarette [10]. The observed high CO levels are correlated with the burning of charcoal [12]. The presence of heavy metals in WPS has been proven to cause lung inflammation, oxidative stress, and, more importantly, increase the risk of chronic obstructive pulmonary disease (COPD) [13,14]. An epidemiological study has revealed that WPS is linked to a higher incidence of emphysema and chronic bronchitis, even after smoking cessation [15]. Moreover, in a cross-sectional study, about 70% of WPS users showed abnormal lung function and an increased risk of COPD [16]. We have also shown experimentally that acute, sub-chronic, and chronic exposure to WPS results in oxidative stress, inflammation, and augmented airway resistance, all of which are linked to a decline in lung function [17,18].

It has been reported that regular U.S. adult WPS users are predominantly non-daily users [19]. Inoue-Choi et al. observed increased mortality risks among non-daily smokers who smoked just 6 to 10 cigarettes per month [19]. We have recently shown that non-daily exposure of mice to WPS is associated with significant effects on thrombosis, endothelial alterations, oxidative stress, blood pressure, and DNA damage in the heart [20,21]. However, to our knowledge, the present study is the first experimental comparative investigation that aims: (1) to estimate the impact of O-WPS versus R-WPS on the histopathology of the lung, (2) to investigate their effects on inflammatory markers and chemokines in the lung, bronchoalveolar lavage fluid (BALF), and plasma as well as oxidative stress, genotoxicity, and mitochondrial dysfunction, and (3) to explore the possible involvement of the mitogen-activated protein kinases (MAPKs) (p-JNK/JNK, p-ERK/ERK, and p-p38/p38) ratios.

## 2. Results

### 2.1. Lung Histopathology

**Figure 1:**
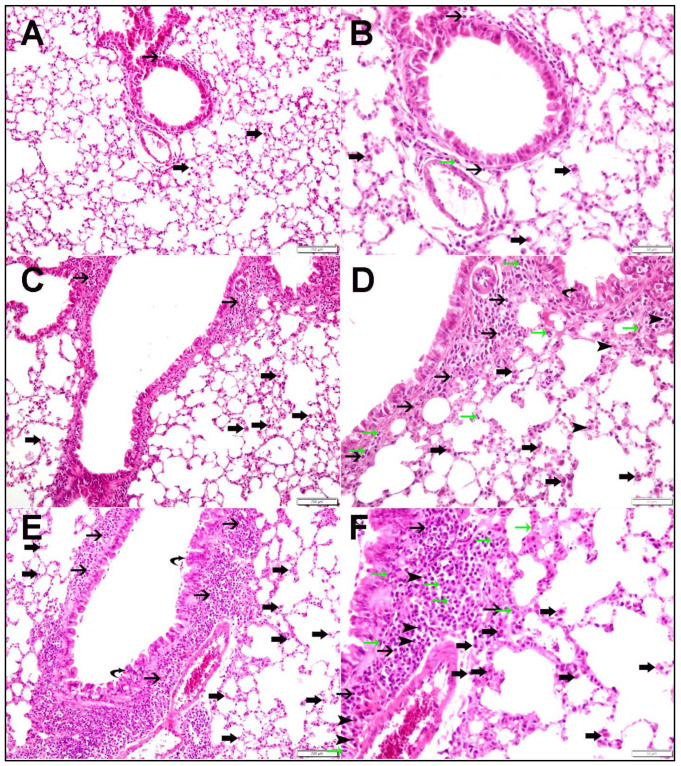
H&E-stained sections of lung tissues taken from mice that were either regularly exposed to WPS (R-WPS), occasionally exposed to waterpipe smoke (O-WPS), or exposed to air (control) for six months. (**A**,**B**) Lung sections obtained from the control group show normal lung architecture and histology, with few lymphocytes seen in peribronchiolar areas (thin arrow) and alveolar macrophages (thick arrow). (**C**,**D**) Lung sections obtained from the O-WPS group show lung tissue with peribronchiolar chronic inflammatory cells consisting of lymphocytes (thin arrow), plasma cells (arrowhead), alveolar macrophages (thick arrow), and neutrophil polymorphs (green arrow). There are also focal syncytial changes in the lining bronchiolar epithelium (curved arrow). (**E**,**F**) Lung sections obtained from the R-WPS group show lung tissue with peribronchiolar chronic inflammatory cells consisting of lymphocytes (thin arrows), plasma cells (arrowheads), alveolar macrophages (thick arrows), and neutrophil polymorphs (green arrows). Moreover, focal syncytial changes of the lining bronchiolar epithelium (curved arrow). (n = 6 in each group). Scale bar in (**A**,**C**,**E**) = 200 µm and scale bar in (**B**,**D**,**F**) = 50 µm.

Figure 1 shows H&E-stained lung sections from mice exposed to air, O-WPS, or R-WPS. Air-exposed mice displayed normal lung architecture with a few lymphocytes in the peribronchiolar regions (Figure 1A,B). O-WPS exposure significantly increased the number of peribronchiolar inflammatory cells, including lymphocytes and alveolar macrophages (Figure 1C,D). R-WPS exposure induced a stronger inflammatory response, with elevated levels of lymphocytes, plasma cells, alveolar macrophages, and neutrophils (Figure 1E,F).

**Figure 2:**
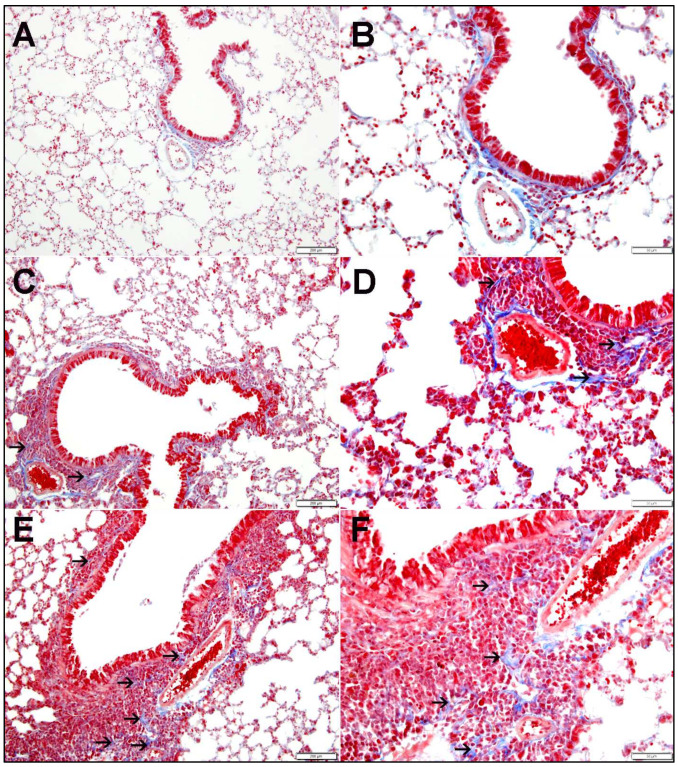
Masson trichrome-stained lung tissue sections of mice exposed to occasional waterpipe smoke (O-WPS), regular WPS (R-WPS), or air for six months are shown in the light microscopy sections. (**A**,**B**) Lung samples from the control group show normal lung architecture and histology, with no signs of fibrosis. (**C**,**D**) Lung sections obtained from the O-WPS group show lung tissue with peribronchiolar inflammatory cells and focal mild fibrous tissue (thin arrow) within the peribronchiolar inflammatory cells. (**E**,**F**) Lung sections obtained from the R-WPS group show lung tissue with peribronchiolar inflammatory cells and focal mild fibrous tissue (thin arrow) within the peribronchiolar inflammatory cells. Scale bar in (**A**,**C**,**E**) = 200 µm and scale bar in (**B**,**D**,**F**) = 50 µm.

In the lungs of air-exposed mice, Masson trichrome stain shows preserved tissue architectures (Figure 2A,B). Masson trichrome stain revealed the presence of focal mild deposition of fibrous tissue in the lungs of O-WPS (Figure 2C,D) or R-WPS mice (Figure 2E,F).

**Figure 3:**
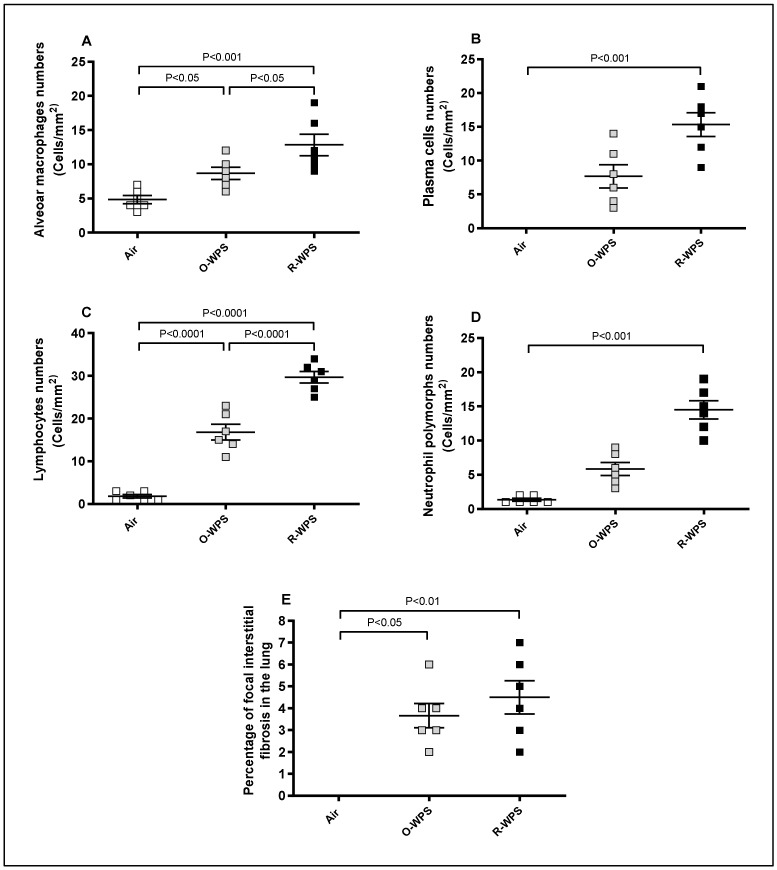
Number of lymphocytes (**A**), plasma cells (**B**), alveolar macrophages (**C**), and neutrophil polymorphs (**D**) quantified by morphometric analysis and focal interstitial fibrosis (**E**) in the lung tissues of mice after exposure to air (control), occasional waterpipe smoke (O-WPS), or regular WPS (R-WPS) for 6 months. Data are presented as the mean ± SEM (n = 6).

Morphometric analysis in Figure 3 demonstrated that O-WPS significantly increased the number of alveolar macrophages (Figure 3A, *p* < 0.05) and lymphocytes (Figure 3C, *p* < 0.0001). R-WPS exposure significantly increased the number of alveolar macrophages (Figure 3A, *p* < 0.001), plasma cells (Figure 3B, *p* < 0.001), lymphocytes (Figure 3C, *p* < 0.0001), and neutrophils (Figure 3D, *p* < 0.001). Both O-WPS and R-WPS increased focal interstitial fibrosis (*p* < 0.05 and *p* < 0.001, respectively) (Figure 3E). Moreover, no perialveolar fibrosis was observed in either the O-WPS or R-WPS groups.

### 2.2. Cell Counts in the BALF

**Figure 4:**
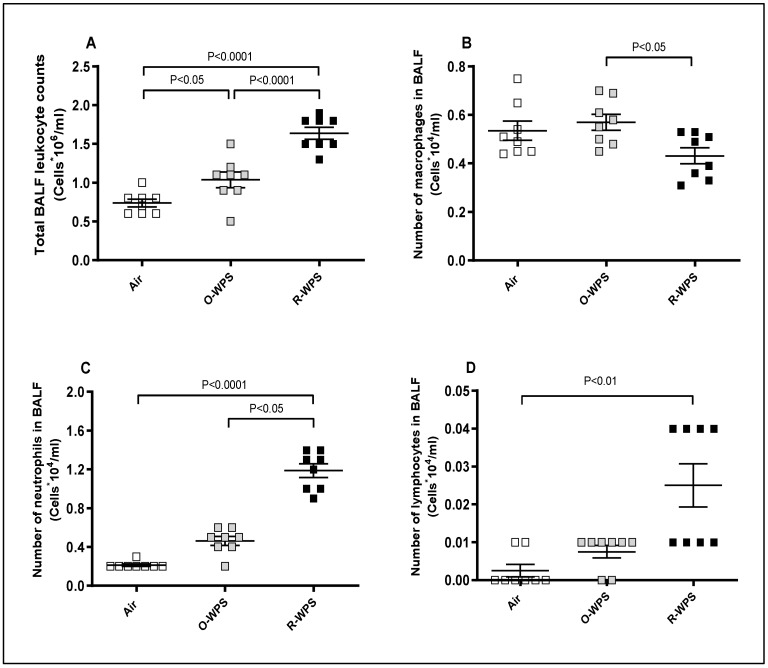
Total leucocytes count (**A**), macrophages (**B**), neutrophils (**C**), and lymphocytes (**D**) in bronchoalveolar lavage fluid (BALF) of mice after exposure to air (control), occasional waterpipe smoke (O-WPS), or regular WPS (R-WPS) for 6 months exposure period to. Data are mean ± SEM (n = 6).

Cell counts in the BALF (Figure 4) revealed that O-WPS significantly increased total leukocyte counts (Figure 4A, *p* < 0.05), but changes in macrophages, neutrophils, and lymphocyte numbers were not significant. However, R-WPS caused a significant increase in the total leukocyte count (Figure 4A, *p* < 0.0001) and the number of neutrophils (Figure 4C, *p* < 0.0001) and lymphocytes (Figure 4D, *p* < 0.01).

### 2.3. Concentrations of CXCL1, CXCL2 and CCL2 in Lung Tissue Homogenates

**Figure 5:**
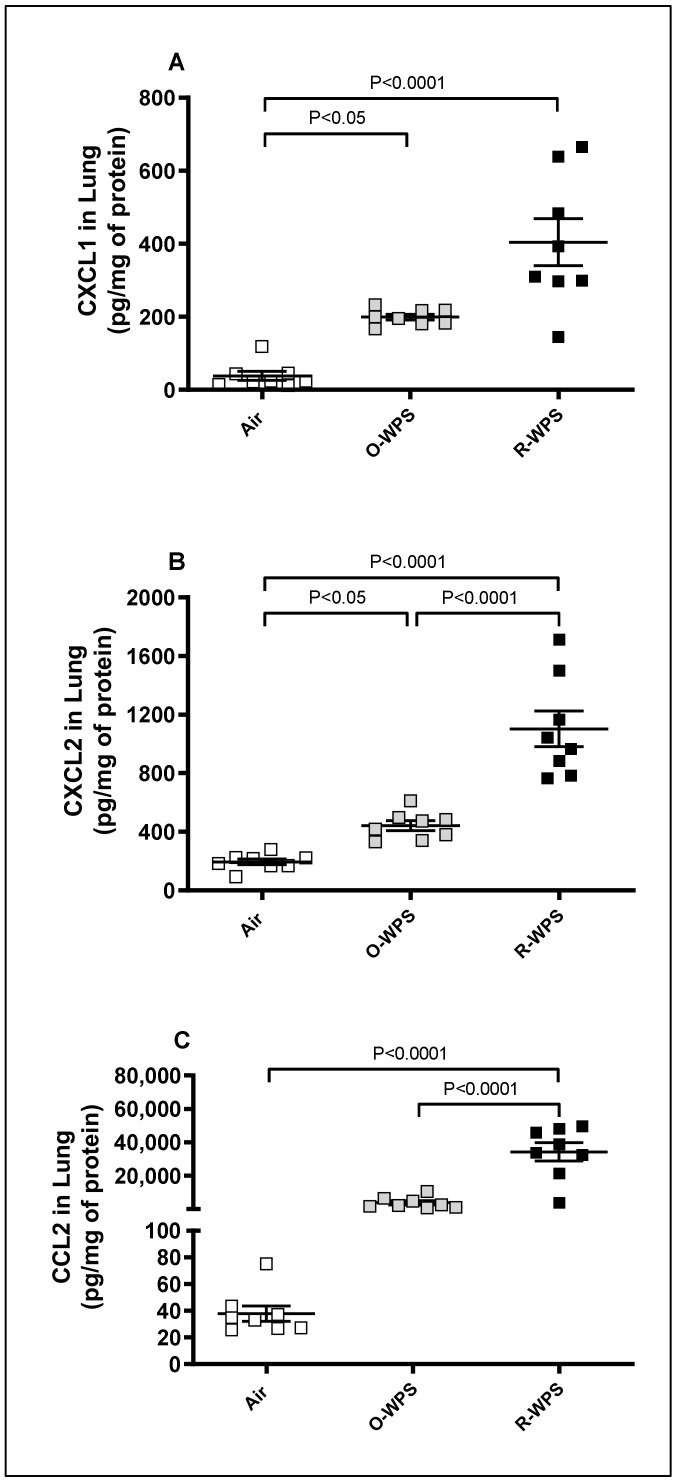
Chemokine CXCL1 (**A**), CXCL2 (**B**), and CCL2 (**C**) concentrations in lung homogenates following exposure to air (control), occasional waterpipe smoke (O-WPS), or regular WPS (R-WPS) for 6 months exposure period. Data are presented as the mean ± SEM (n = 7–8).

The measurement of the concentrations of chemokines CXCL1, CXCL2, and CCL2 in lung homogenates following exposure to O-WPS revealed a significant increase in CXCL1 (*p* < 0.05) and CXCL2 (*p* < 0.05) concentrations compared with the air-exposed group. On the other hand, compared with the control group, R-WPS induced a significant augmentation in the concentration of the three chemokines, CXCL1 (*p* < 0.0001), CXCL2 (*p* < 0.0001), and CCL2 (*p* < 0.0001) (Figure 5).

### 2.4. Concentrations of CXCL1, CXCL2 and CCL2 in BALF

**Figure 6:**
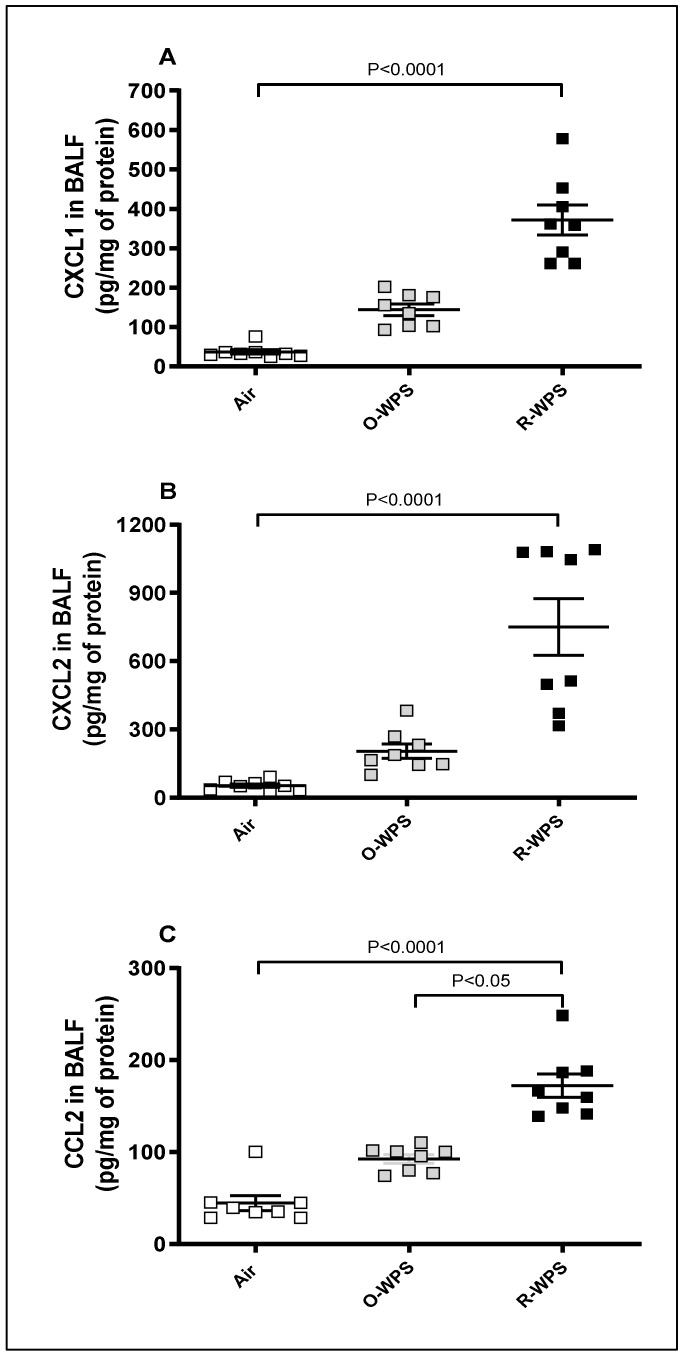
Chemokines CXCL1 (**A**), CXCL2 (**B**), and CCL2 (**C**) concentrations in bronchoalveolar lavage fluid (BALF) following exposure to air (control), occasional waterpipe smoke (O-WPS), or regular WPS (R-WPS) for 6 months exposure period. Data are presented as the mean ± SEM (n = 7–8).

Figure 6 shows that exposure to O-WPS did not have a pronounced impact on CXCL1, CXCL2, and CCL2. In contrast, exposure to R-WPS caused a significant elevation in CXCL1 (*p* < 0.0001, Figure 6A), CXCL2 (*p* < 0.0001, Figure 6B), and CCL2 (*p* < 0.0001, Figure 6C).

### 2.5. Concentrations of CXCL1, CXCL2 and CCL2 in Plasma

**Figure 7:**
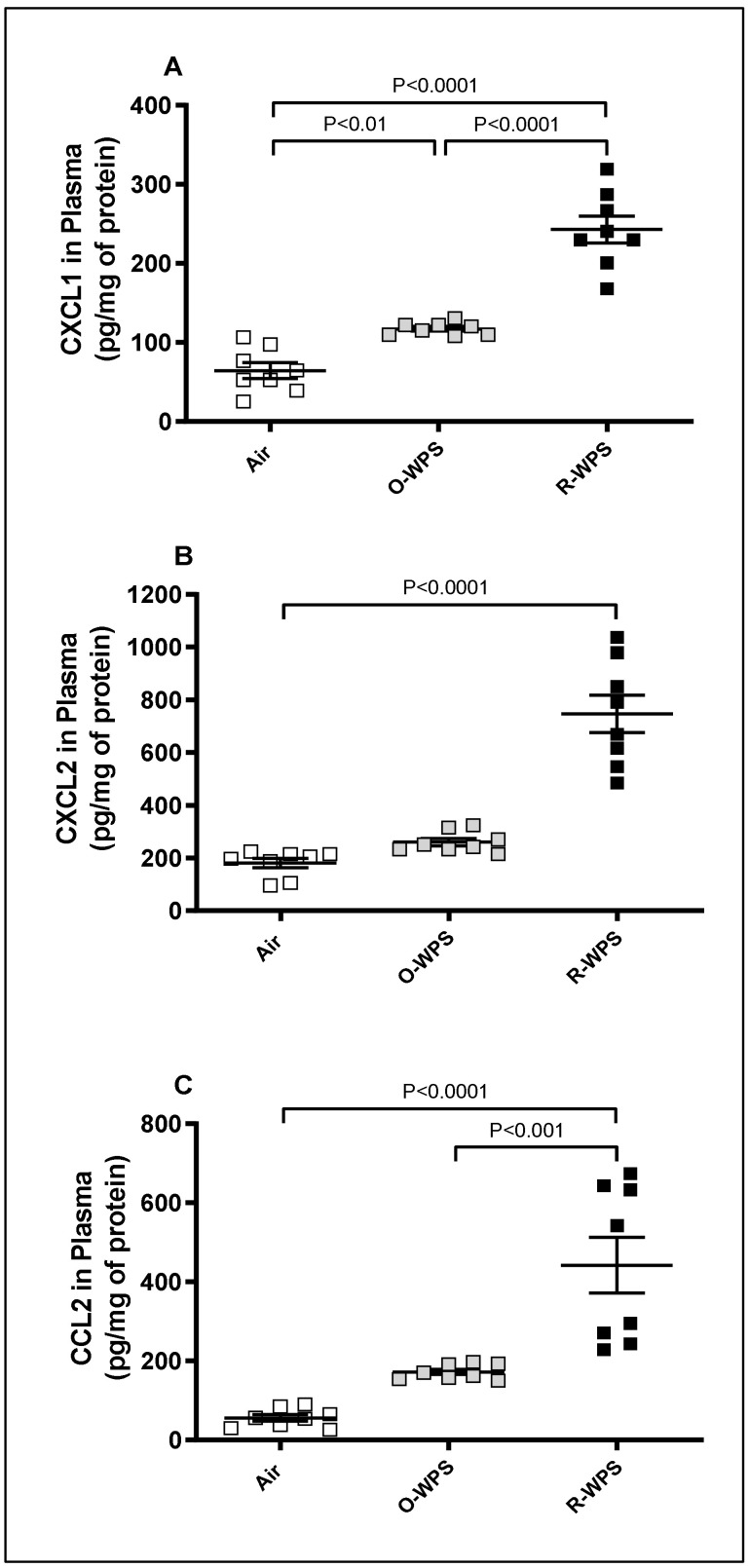
Chemokines CXCL1 (**A**), CXCL2 (**B**), and CCL2 (**C**) concentrations in plasma following exposure to air (control), occasional waterpipe smoke (O-WPS), or regular WPS (R-WPS) for 6 months exposure period. Data are presented as the mean ± SEM (n = 7–8).

The assessment of chemokines CXCL1 (Figure 7A), CXCL2 (Figure 7B), and CCL2 (Figure 7C) in the plasma revealed that exposure to either O-WPS or R-WPS results in a significant augmentation in the concentration of CXCL1 (*p* < 0.01) and (*p* < 0.0001), respectively. Compared with the air group, chronic exposure to R-WPS led to a significant increase in the concentrations of CXCL2 (*p* < 0.0001) and CCL2 (*p* < 0.0001).

### 2.6. Levels of MMP-9, MPO, and LDH in Lung Tissue Homogenates

**Figure 8:**
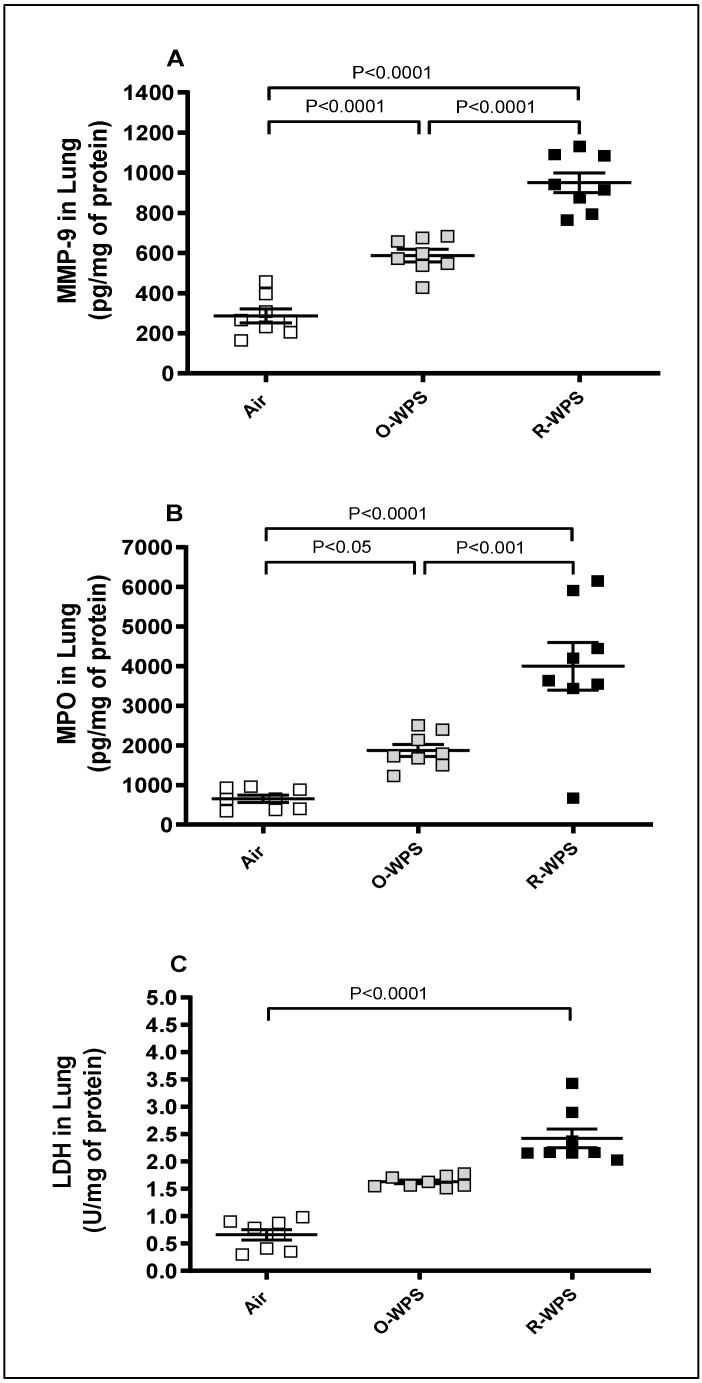
Matrix metalloproteinase-9 (MMP-9, (**A**)), myeloperoxidase (MPO, (**B**)), and lactate dehydrogenase (LDH, (**C**)) levels in lung homogenates following exposure to air (control) or occasional waterpipe smoke (O-WPS) or regular WPS (R-WPS) for 6 months exposure period. Data are presented as the mean ± SEM (n = 7–8).

As shown in Figure 8A, the concentrations of MMP-9 were found to be significantly augmented following exposure to either O-WPS (*p* < 0.0001) or R-WPS (*p* < 0.0001). Likewise, both O-WPS (*p* < 0.05) and R-WPS (*p* < 0.0001) induced a marked elevation in MPO levels (Figure 5B). Moreover, as illustrated in Figure 5C, the activity of LDH was significantly higher in the R-WPS group (*p* < 0.0001) than in the control group.

### 2.7. Concentrations of IL1β, IL6 and TNFα in Lung Tissue Homogenates

**Figure 9:**
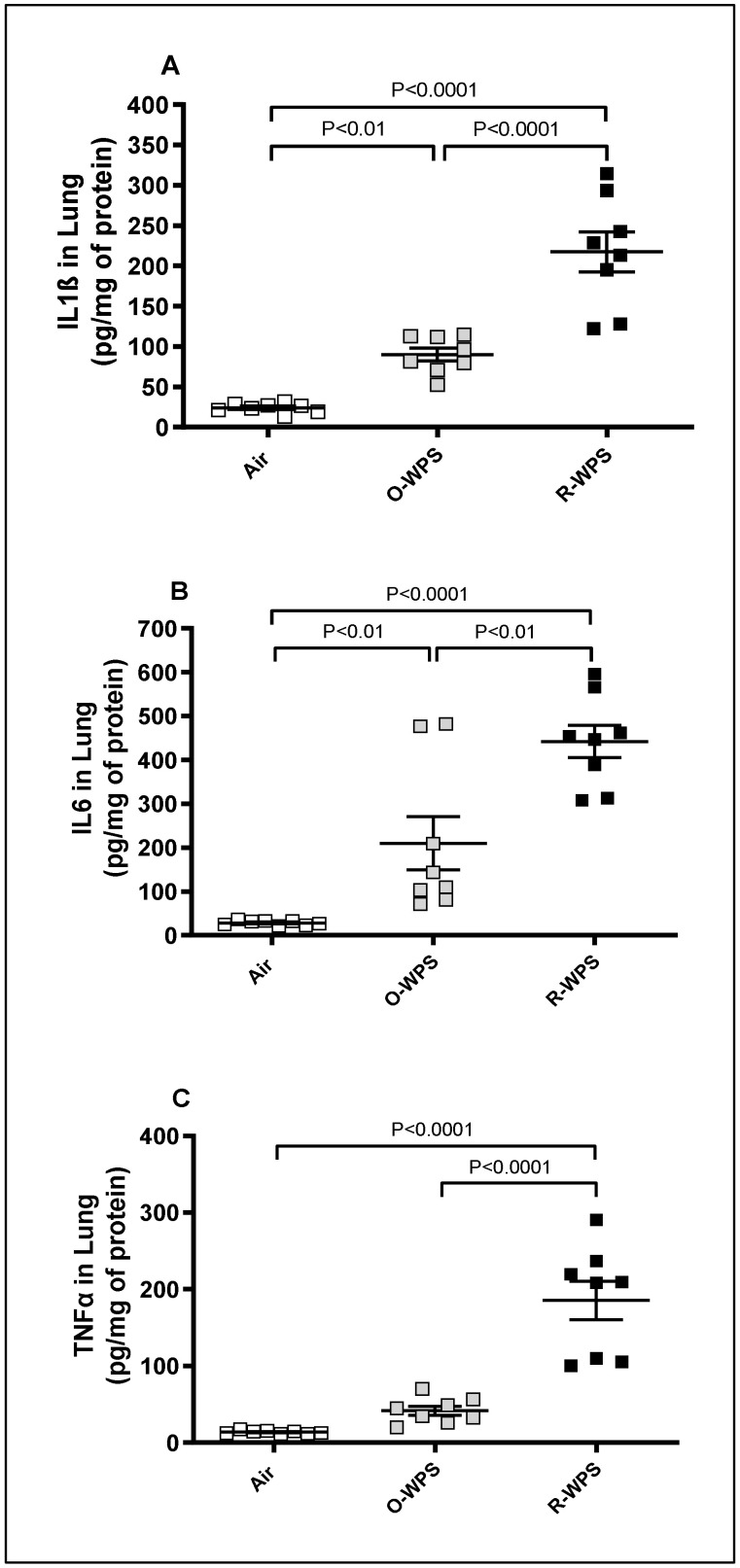
Interleukin (IL)-1β (**A**), IL-6 (**B**), and tumor necrosis factor α (TNFα, (**C**)) concentrations in lung homogenates following exposure to air (control), occasional waterpipe smoke (O-WPS), or regular WPS (R-WPS) for 6 months exposure period. Data are presented as the mean ± SEM (n = 7–8).

Figure 9 depicts the impact of 6 months of exposure to either O-WPS or R-WPS on the concentrations of inflammatory cytokines comprising IL1β, IL-6, and TNF-α in lung tissue homogenates. Compared with the air-exposed group, O-WPS triggered a significant increase in IL1β (*p* < 0.01) and IL-6 (*p* < 0.01), but not TNF-α. However, regular exposure to WPS caused a significant elevation in the concentrations of IL1β (*p* < 0.0001), IL-6 (*p* < 0.0001), and TNF-α (*p* < 0.0001) compared with the air-exposed group. Furthermore, the concentrations of all these proinflammatory cytokines were significantly augmented in mice exposed to R-WPS compared with those exposed to O-WPS (*p* < 0.0001-*p* < 0.01).

### 2.8. Concentrations of IL1β, IL6 and TNFα in BALF

**Figure 10:**
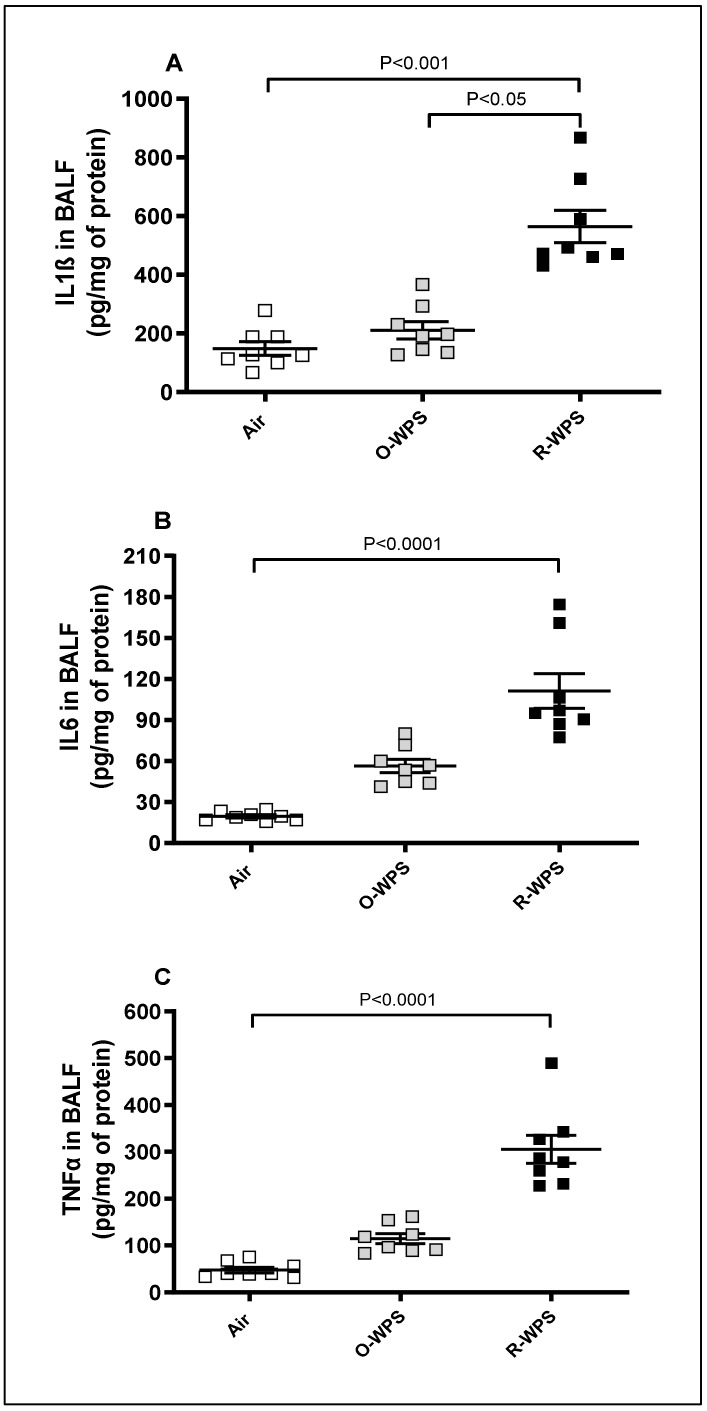
Interleukin (IL)-1β (**A**), IL-6 (**B**), and tumor necrosis factor α (TNFα, (**C**)) concentrations in bronchoalveolar lavage fluid (BALF) following exposure to air (control), occasional waterpipe smoke (O-WPS), or regular WPS (R-WPS) for 6 months exposure period. Data are presented as the mean ± SEM (n = 7–8).

The concentrations of IL1β (*p* < 0.001), IL6 (*p* < 0.0001), and TNFα (*p* < 0.0001) in BALF were significantly augmented following chronic exposure to R-WPS. O-WPS exposure caused an increase in the three proinflammatory cytokines but did not reach significant levels (Figure 10).

### 2.9. Concentrations of IL1β, IL6 and TNFα in Plasma

**Figure 11:**
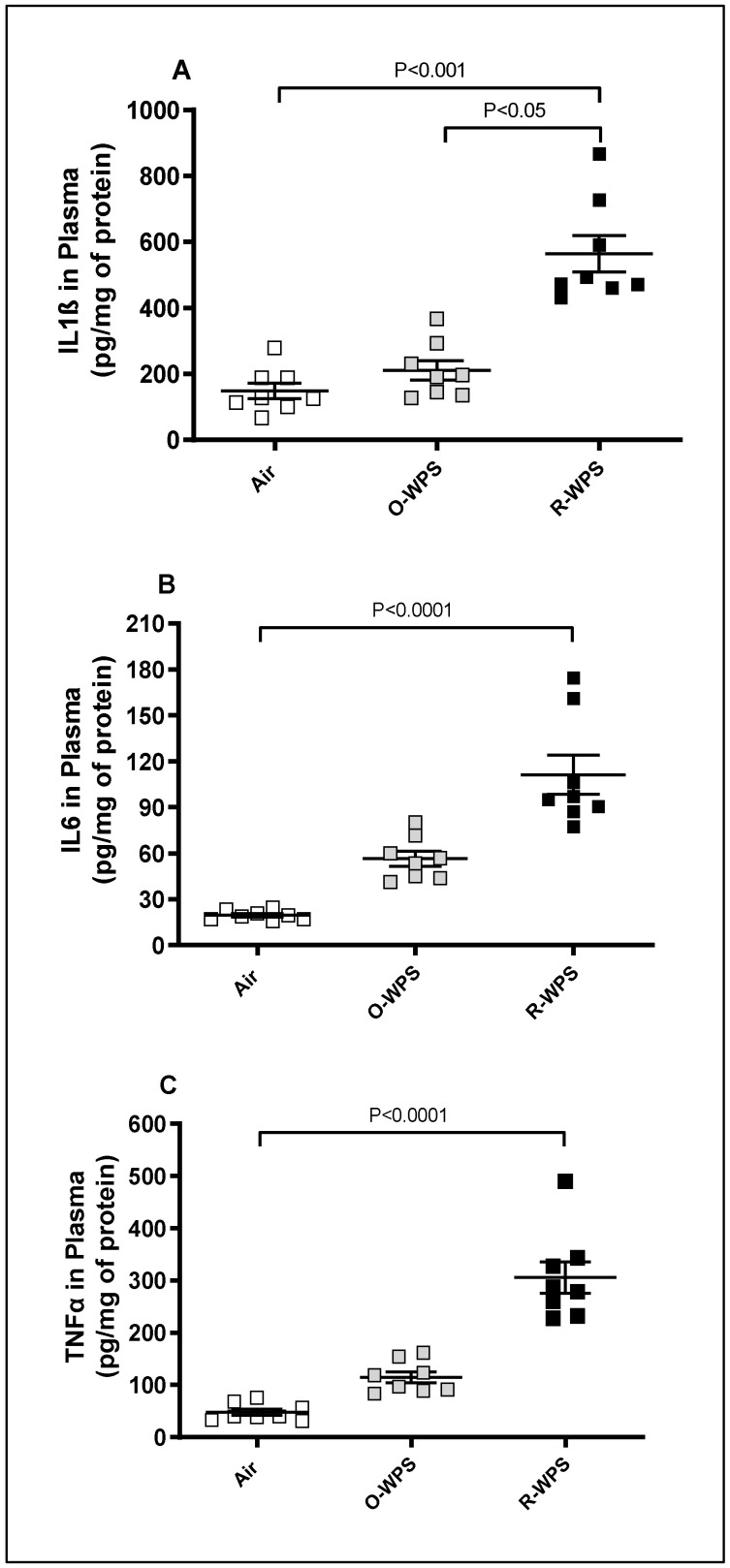
Interleukin (IL)-1β (**A**), IL-6 (**B**), and tumor necrosis factor α (TNFα, (**C**)) concentrations in plasma following exposure to air (control), occasional waterpipe smoke (O-WPS), or regular WPS (R-WPS) for 6 months exposure period. Data are presented as the mean ± SEM (n = 7–8).

The measurement of proinflammatory cytokines IL1β (Figure 11A), IL6 (Figure 11B), and TNFα (Figure 11C) in the plasma revealed that chronic inhalation of R-WPS induced a significant elevation in the concentration of IL1β (*p* < 0.001), IL6 (*p* < 0.0001), and TNFα (*p* < 0.0001) compared with the air group. Exposure to O-WPS did not significantly increase the concentrations of these three proinflammatory cytokines.

### 2.10. Levels of TBARS and SOD in Lung Tissue Homogenates 

**Figure 12:**
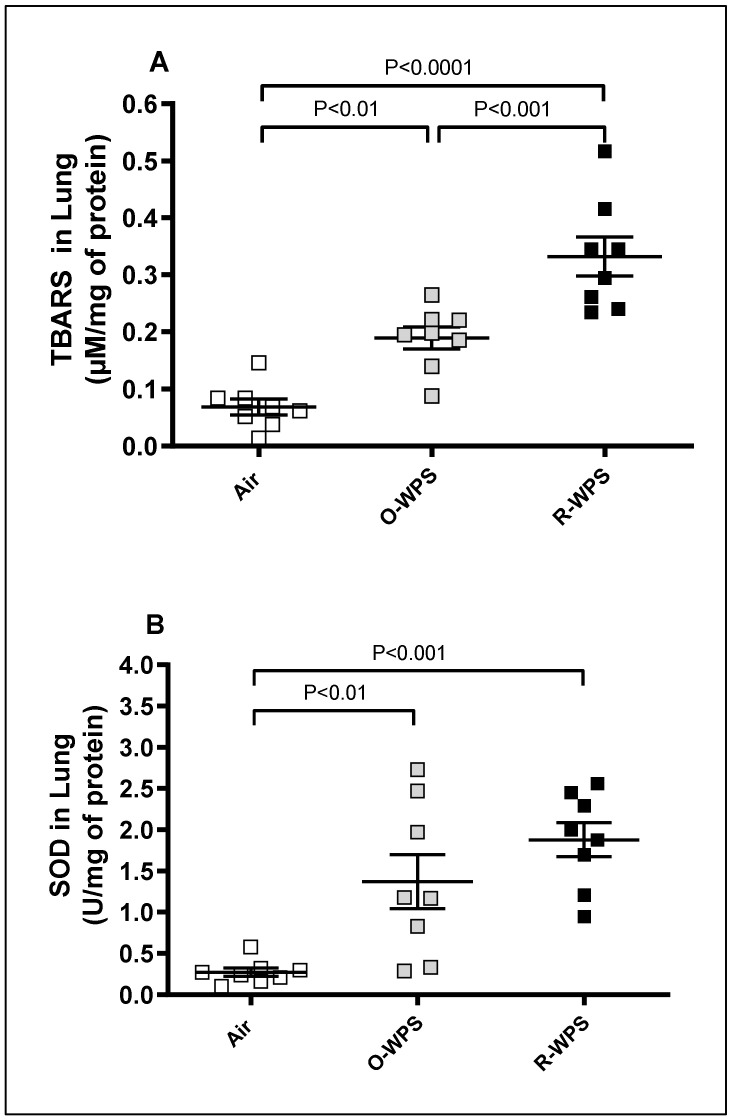
Thiobarbituric acid reactive substances (TBARS, (**A**)) and superoxide dismutase (SOD, (**B**)) levels in lung homogenates following exposure to air (control), occasional waterpipe smoke (O-WPS), or regular WPS (R-WPS) for 6 months exposure period. Data are presented as the mean ± SEM (n = 7–8).

In comparison with air exposure, our results show that the concentration of TBARS and the activity of SOD were significantly augmented in O-WPS (*p* < 0.01) and (*p* < 0.01), respectively. Similarly, R-WPS induced a significant increase in the concentration of TBARS (*p* < 0.0001) and SOD (*p* < 0.001) (Figure 12). Moreover, the concentration of TBARS in mice exposed to the R-WPS group was higher than that recorded in O-WPS (*p* < 0.001).

### 2.11. DNA Migration and Concentrations of 8-OHdG in Lung Tissue Homogenates

**Figure 13:**
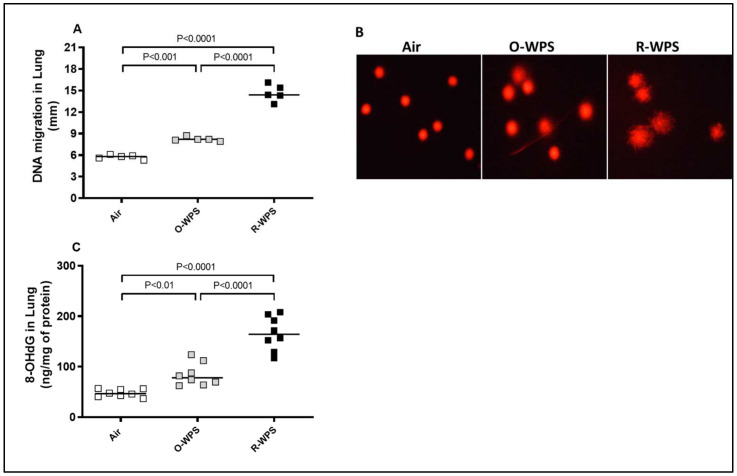
DNA migration (**A**), representative images of single-cell gel electrophoresis (comet assay) showing DNA damage (magnification 40×) (**B**), and concentration of 8-Hydroxy-2′-deoxyguanosine (8-OHdG, (**C**)) in lung homogenates following exposure to air (control), occasional waterpipe smoke (O-WPS), or regular WPS (R-WPS) for 6 months exposure period. Data are presented as the mean ± SEM (n = 7–8).

Figure 13A illustrates that, compared with the air-exposed group, chronic exposure to either O-WPS or R-WPS induced a significant (*p*< 0.001 and *p*< 0.0001, respectively) augmentation of DNA migration, illustrating the occurrence of DNA damage. Furthermore, there was more DNA migration in R-WPS than in O-WPS (*p*< 0.0001). Figure 13B shows representative images of a single-cell gel electrophoresis (Comet assay) indicating DNA damage, in terms of fragmentation, quantified by the measurement of the length of the DNA migration (i.e., diameter of the nucleus plus migrated DNA). The latter results showed more DNA migration in mice exposed to O-WPS and R-WPS compared with the control group and in R-WPS compared with O-WPS. As shown in Figure 13C, the concentration of the oxidative DNA damage marker, 8-OHdG, was significantly elevated in the lungs of mice exposed to either O-WPS (*p*< 0.01) or R-WPS (*p*< 0.0001) for 6 months compared with the air-exposed group. In addition, there was a significant difference between the O-WPS and R-WPS groups (*p*< 0.0001).

### 2.12. Mitochondrial Complexes I (A), II and III (B), IV (C) Activities in Lung Tissue Homogenates

**Figure 14:**
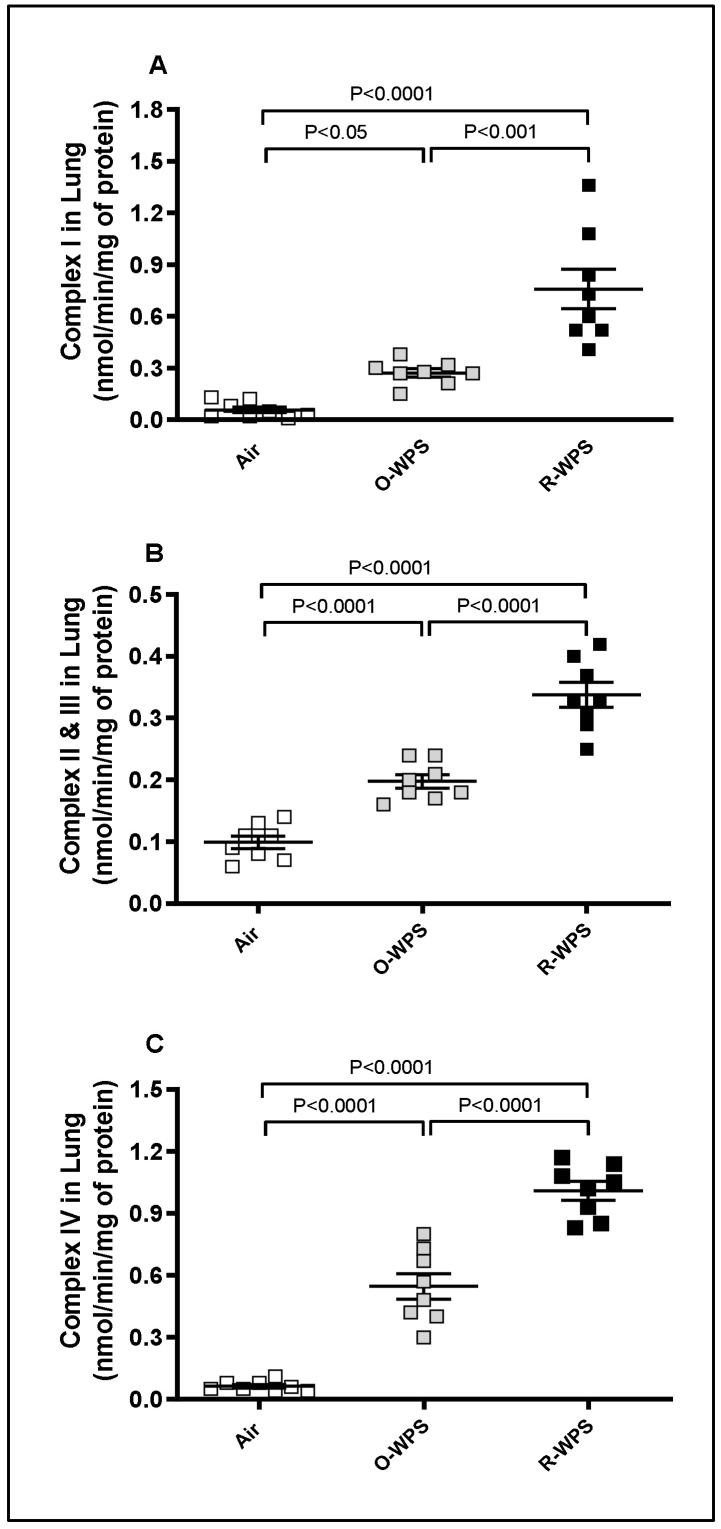
Mitochondrial complex I (**A**), II and III (**B**), and IV (**C**) activities in lung homogenates following exposure to air (control), occasional waterpipe smoke (O-WPS), or regular WPS (R-WPS) for a 6 months exposure period. Data are presented as the mean ± SEM (n = 7–8).

In comparison with the air-exposed group, O-WPS inhalation induced a significant increase in the lung activities of mitochondrial complexes I (*p* < 0.05), II and III (*p* < 0.0001), and IV (*p* < 0.0001). Similarly, all complexes were augmented following exposure to R-WPS compared with either control (*p* < 0.0001) (Figure 14).

### 2.13. Expression of p-JNK/JNK, p-ERK/ERK and p-p38/p38 Ratios in Lung Tissue Homogenates

**Figure 15:**
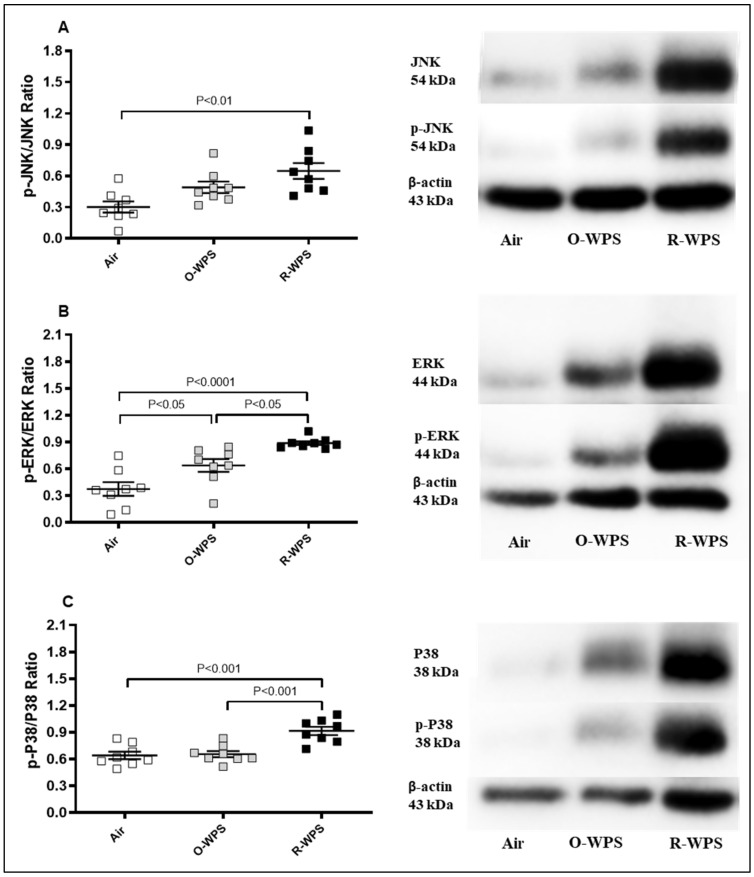
Western blot analysis of the ratios of phosphorylated (phospho) c-Jun NH2-terminal kinase (p-JNK/JNK, (**A**)), extracellular signal-regulated kinase (p-ERK/ERK, (**B**)), and p38 mitogen-activated protein kinases (p-p38/p38, (**C**)) in lung homogenates following exposure to air (control), occasional waterpipe smoke (O-WPS), or regular WPS (R-WPS) for 6 months exposure period. Data are presented as the mean ± SEM (n = 7–8).

The expression of p-JNK/JNK, p-ERK/ERK, and p-p38/p38 ratios was assessed using the Western blot technique, which is shown in Figure 15. Compared with the air-exposed group, R-WPS exposure induced a significant increase in the expression of p-JNK/JNK (*p* < 0.01), p-ERK/ERK (*p* < 0.0001), and p-p38/p38 (*p* < 0.001) ratios. O-WPS exposure induced a significant increase only in the p-ERK/ERK ratio (*p* < 0.0001).

## 3. Discussion

Several studies conducted by our research group and others have provided compelling evidence regarding the detrimental impact of R-WPS exposure on the lungs [18,22,23,24]. However, as far as we are aware, there is a lack of experimental data regarding the effects of O-WPS on such organ. Thus, in the current investigation, we aimed to assess the histological and biochemical changes in the lungs resulting from exposure to O-WPS versus R-WPS.

The findings of the current study demonstrated that long-term exposure to either O-WPS or R-WPS elicited infiltration in the lung parenchyma of a plethora of inflammatory cells, consisting of lymphocytes and alveolar macrophages, and caused focal interstitial fibrosis. Additionally, this study showed that exposure to O-WPS or R-WPS triggered a significant increase in the levels of chemokines and proinflammatory cytokines in the lung, BALF, and plasma, as well as augmentation in the levels of lung injury enzymes (MMP-9, MPO, and LDH), oxidative stress (TBARS and SOD), markers of genotoxicity, and mitochondrial dysfunction. Furthermore, we observed a significant elevation in MAPK ratios (p-JNK/JNK, p-ERK/ERK, and p-p38/p38) in the lung.

Previous research, including our own, has shown that exposure to WPS results in a reduction in alveoli count, thickening of the alveolar septa, and enlargement of the alveolar interstitial space, accompanied by an influx of inflammatory cells such as neutrophils, macrophages, and lymphocytes [18,23,25]. Our present data indicate that prolonged exposure to either O-WPS or R-WPS caused increased infiltration of lymphocytes and alveolar macrophages and induction of interstitial fibrosis in the lung tissue. In addition, we observed infiltration of neutrophil polymorphs and plasma cells in the R-WPS group. The BALF analysis showed that exposure to either O-WPS or R-WPS caused a significant augmentation in the total leucocyte count. Additionally, R-WPS inhalation induced a significant increase in the number of neutrophils and lymphocytes in the BALF. These findings clearly show that irrespective of the mode of exposure, WPS was able to trigger damaging effects in the lungs. Our present findings align with previous studies showing that CS, comprising secondhand exposure, induces infiltration and recruitment of macrophages in the lungs of mice [26,27]. Moreover, CS exposure in both humans and experimental models has been linked to increased neutrophil counts in BALF and total lymphocyte counts in the blood [28,29,30,31].

It is well established that infiltration of inflammatory cells and their recruitment are facilitated by chemokines [32]. Here, we observed that both O-WPS and R-WPS induced an increase in the concentrations of the chemokines CXCL1 and CXCL2 in lung homogenates. However, only R-WPS exposure resulted in the augmentation of CXCL1, CXCL2, and CCL2 concentrations in the lungs, BALF, and plasma. We have shown recently in the heart that exposure to both regimens causes an elevation in the levels of CXCL1 chemokine, and R-WPS exposure leads to an increase in CCL2 [21]. Consistent with our findings, it has been reported that WPS condensate enhances the expression of inflammatory response genes, including CCL2 chemokine, IL-1β, and IL-6, in lung cells [33]. Furthermore, exposure to CS increased the expression of ileal CXCL2 in mice [33,34], while an in vitro study demonstrated that prolonged CS exposure induced increased production of CXCL1 and CXCL2 in alveolar epithelial cells [35].

MMP-9 is a proteolytic enzyme that plays a crucial role in tissue remodeling and has been found to be involved in the development of airway inflammation [36]. The alveolar macrophages are considered a major source of MMP-9 production [37,38]. Myeloperoxidase (MPO) is a powerful oxidizing agent that is stored in neutrophils. During severe inflammation, neutrophils undergo secondary necrosis, leading to the release of MPO, which can impair local lung cells [39]. In this study, the significant increases in the levels of MPO and MMP-9 after long-term inhalation of O-WPS and R-WPS were linked to the observed influx of inflammatory cells into the lung parenchyma. Supporting our findings, numerous studies have reported increased levels of MPO and MMP-9 following exposure to WPS [18,25,40,41]. Furthermore, both in vivo and in vitro studies have indicated that exposure to CS significantly elevated these enzymes [42,43,44]. Clinically, it has been found that cigarette smokers exhibited markedly higher serum concentrations of MMP-9 and CRP compared to non-smokers [45]. LDH, an enzyme that facilitates the conversion of lactate to pyruvate, elevated LDH activity serves as an indicator of tissue damage [46]. The presented data showed greater LDH levels following exposure to R-WPS than in the control group. We have previously demonstrated increased LDH activity in mice following exposure to WPS [18,47]. Similarly, exposure to CS causes a significant elevation in LDH activity in rat lungs [48,49]. Moreover, treating A549 cells with the CS extract resulted in a notable increase in LDH levels [50].

Our data indicated that the concentrations of IL1β, IL6, and TNFα were significantly elevated in the lungs, BALF, and plasma after exposure to R-WPS. Additionally, O-WPS exposure caused a notable increase in IL1β and IL6 in the lung. Our current findings are congruent with our previous and other studies that have reported that exposure to WPS increases the levels of proinflammatory cytokines, including IL-1β, IL-6, and TNF-α [13,18,22,23,24,51,52]. Furthermore, both in vivo and in vitro studies have demonstrated that CS elevated the expression of proinflammatory mediators, including IL-8, TNF-α, and MMP-9, in rat lungs and macrophages [53]. The effect of WPS on inflammatory markers in lung tissue could be attributed to the extensive infiltration of different inflammatory cells documented in this study. In addition, it has been suggested that the enzyme MPO, secreted by neutrophils and monocytes, is essential for innate immunity and can worsen the inflammatory response [54,55].

Numerous inflammatory cells, including lymphocytes, macrophages, and neutrophils, are known to play a role in the pathophysiology of COPD [56]. The recruitment of these cells is thought to be a source of numerous mediators, such as cytokines and reactive oxygen species, which inevitably contribute to oxidative stress in the lungs [57]. The infiltration of inflammatory cells into the lung parenchyma observed in the current study was accompanied by an elevation in the levels of oxidative stress markers, TBARS, and SOD following exposure to O-WPS or R-WPS.

The results of this study are in line with previous clinical and experimental studies, which demonstrated that WPS exposure causes lung inflammation and oxidative stress [25,58,59,60]. Additionally, Khabour et al. reported that WPS exposure increases the activity of various antioxidant enzymes, including catalase, glutathione peroxidase, and superoxide dismutase, in the lung [13].

Clinical evidence has shown that exposure to WPS causes DNA damage, oxidative stress, and the suppression of DNA repair gene expression in humans [61]. Similarly, previous studies have reported that WPS leads to DNA damage in the lungs of mice [22,25,41]. In the current study, we observed that in addition to R-WPS, occasional exposure to WPS one day per week caused a significant elevation in DNA damage, manifested in significant augmentation of DNA migration and 8-OHdG concentration. The latter effect can be linked to the oxidative stress and inflammation recorded in the current study [62]. In addition, the observed DNA damage induced by WPS may be attributed to the chemicals present in WPS that possess a genotoxic effect, such as heavy metals, naphthalene, aldehydes, and carbon monoxide [63].

Our data showed that both modes of exposure to WPS induced mitochondrial dysfunction, as evidenced by an increase in the activity of mitochondrial complexes I, II, III, and IV. Consistent with the latter, our recent findings indicate that WPS inhalation causes mitochondrial dysfunction in the heart [21,64]. Similarly, cigarette smokers exhibit increased mitochondrial DNA alterations in buccal cells compared to healthy non-smokers [65]. Mitochondrial hyperfusion has also been observed in mouse alveolar epithelial cells following exposure to low doses of the CS extract [66]. Additionally, CS exposure has been shown to disrupt the balance between mitochondrial fusion and fission, elevate oxidative stress, and induce morphological changes in the mitochondria of primary rat lung microvascular endothelial cells [67].

Three primary MAPKs (JNK, ERK, and p38) are governed by separate extracellular signal transduction pathways in the nucleus, which leads to gene regulation [68]. MAPKs influence cell physiology in multiple ways, including differentiation, cell growth, and programmed cell death [69,70]. More importantly, JNK, ERK, and p38 are found to play critical regulatory roles in the production of proinflammatory cytokines (e.g., TNF-α, IL-1, IL-2, and IL-6) and downstream signaling related to inflammation [70]. Our results showed that while O-WPS exposure induced a significant increase only in the p-ERK/ERK ratio, R-WPS markedly increased the ratios of p-JNK/JNK, p-ERK/ERK, and p-p38/p38 in lung homogenates compared with the controls.

These findings suggest that p-JNK, p-ERK, and p-p38 signaling cooperatively contribute to inflammation induced by WPS exposure. Supporting our findings, exposure of myotubes to CS extract has been shown to activate phosphorylated MAPKs, including p38, JNK, and ERK1/2 [71]. Similarly, endothelial cells exposed to tobacco smoke condensate exhibited significant activation of MAPKs, particularly ERK and p38 [72]. Furthermore, it has been reported that the CS extract triggers an extrinsic apoptotic pathway in human erythrocytes, mediated through the p38 signaling pathway [73]. Most notably, MAPK data seem to suggest that depending on the mode of exposure, there are some differences in the mechanisms underpinning the pulmonary toxicity induced by WPS inhalation, and that additional work is warranted to address this point.

The limitations of this study include the use of only male animals, which may have affected the generalizability of the findings. Additionally, the presence of 8-OHdG could be more comprehensively demonstrated through immunostaining or Western blotting. Moreover, techniques such as flow cytometry and immunohistochemistry can provide further insights by tracking the migration of neutrophils, macrophages, or other immune cells into the alveolar or lung interstitial space. Furthermore, assessment of the gene expression of inflammatory chemokines and cytokines would enhance our understanding of the inflammatory response.

The present study provides, for the first time, clear evidence that WPS exposure, regardless of its frequency (regular or occasional), can lead to a range of detrimental pathophysiological effects in the lungs. These effects include infiltration of immune cells into the lung parenchyma, inflammation, oxidative stress, genotoxicity, and mitochondrial dysfunction. This study demonstrated that these effects were linked to the intracellular activation of key signaling pathways, specifically JNK, ERK, and p38. These findings underscore the need for increased awareness and education regarding serious health risks related to waterpipe smoking, regardless of the frequency of use. 

## 4. Materials and Methods

### 4.1. Animals and WPS Exposure

Male BALB/c mice, aged between 6 and 8 weeks and weighing 20 to 25 g (sourced from Taconic Farms Inc., Germantown, NY, USA), were kept in the central animal facility of the College of Medicine and Health Sciences. They had free access to food and water and were maintained under regulated conditions, including a 12-h light/dark cycle, 60% humidity, and a temperature of 22 ± 1 °C. Following a week of acclimatization to the experimental environment, the mice were randomly divided into three groups: air (control), O-WPS, or R-WPS. The exposure protocol for WPS was in accordance with previously established methods [74,75]. The mice were carefully inserted into restrainers that were attached to a WPS exposure tower. The mice were able to inhale either air or WPS through a nose-only exposure system connected to a waterpipe apparatus (inExpose System; SCIREQ, Montreal, QC, Canada). For each daily session, 10 g of commercially available apple-flavored tobacco was used in the waterpipe head in order to generate WPS, with any leftover tobacco being disposed of at the end of each session [74,75].

Control mice were exposed to air only, with each session lasting 30 min per day. Mice in the R-WPS group were exposed to WPS for 30 min, 5 days per week, over a period of 6 months, while those in the O-WPS group were exposed to WPS for the same duration but only 1 day per week for 6 months [20,21]. A computerized system controls the WPS exposure procedure, administering puffs every minute, including a 2-s WPS inhalation and 58 s of fresh air. The duration of inhalation exposure was restricted to 30 min per day, as documented in prior studies involving both human participants and experimental mice [25,58,75,76,77].

### 4.2. Lung Histopathology

The lungs were extracted, cleaned with ice-cold saline, weighed, and then dried using filter paper. After being placed in a cassette, each lung was fixed for a whole day in 10% neutral formalin. Paraffin sections of 4 μm thickness were stained with hematoxylin and eosin (H&E) [78]. The number of inflammatory cells was quantified and expressed as the number of inflammatory cells per mm^2^. Masson’s trichrome staining was performed to evaluate the extent of fibrosis [79]. The histopathologist who participated in this investigation evaluated the stained sections under light microscopy.

### 4.3. Blood Collection and Biochemical Analysis

Using sodium pentobarbital for anesthesia, a dose of (60 mg/kg) was administered intraperitoneally, and blood was drawn from the inferior vena cava, collected into heparinized tubes, and centrifuged at 900× *g* at 4 °C for 15 min to isolate the plasma. The plasma obtained was stored at −80 °C for future biochemical analyses. Following this, with an overdose of sodium pentobarbital, the mice were euthanized. The lung was then excised, wrapped in aluminum foil, immersed in liquid nitrogen, and stored at −80 °C until further analysis. The preparation of lung homogenates for biochemical analysis was performed as previously detailed [80].

### 4.4. Collection and Analysis of BAL Fluid

The collection and analysis of BALF were conducted as previously described [81,82]. In summary, after exposure to either O-WPS, R-WPS, or air, the mice were euthanized with an overdose of pentobarbital sodium. Their tracheas were cannulated, and the lungs were lavaged three times with 0.7 mL of sterile 0.9% NaCl (totaling 2.1 mL). The retrieved fluid samples were combined, and no significant difference in the volume of fluid collected was noted among the groups. The BALF was then centrifuged at 1000× *g* for 10 min at 4 °C. Cell counts were performed and differential counts were conducted microscopically on cytocentrifuge preparations fixed in methanol and stained with Diff Quick (Dade, Brussels, Belgium). The supernatant was preserved at −80 °C for future analysis.

### 4.5. Measurement of Chemokines (CXCL1, CXCL2 and CCL2), Matrix Metalloproteinase-9 (MMP-9), Myeloperoxidase (MPO), Lactate Dehydrogenase (LDH), Interleukin-1β (IL1β), IL6, Tumor Necrosis Factor α (TNFα) and 8-Hydroxy-2-Deoxyguanosine (8-OHdG) in Lung Homogenates

Using enzyme-linked immunosorbent assay (ELISA) kits, we measured the concentrations of CXCL1 (Lot N° DY453, R&D systems, Minneapolis, MN, USA), CXCL2 (Lot N° DY452, R&D systems, Minneapolis, MN, USA), CCL2 (Lot N° DY479, R&D systems, Minneapolis, MN, USA), MMP-9 (Lot N° DY6718, R&D systems, Minneapolis, MN, USA), MPO (Lot N° DY3667, R&D systems, Minneapolis, MN, USA), IL-1β (Lot N° DY401, R&D systems, Minneapolis, MN, USA), IL6 (Lot N° DY406, R&D systems, Minneapolis, MN, USA), TNFα (Lot N° DY410, R&D systems, Minneapolis, MN, USA), and -OHdG (Lot N° E-EL-0028, R&D systems, Minneapolis, MN, USA).

### 4.6. DNA Damage Assessment in the Lung by COMET Assay

In another group of animals, lung tissues were collected immediately after sacrifice and analyzed for DNA damage using the COMET assay, as previously described [83,84]. The assessment of DNA migration, which includes measuring the nucleus diameter and the extent of migrated DNA, was performed using Axiovision 3.1 image analysis software (Carl Zeiss, Toronto, ON, Canada), following earlier methodologies.

### 4.7. Measurement of Thiobarbituric Acid Reactive Substances (TBARS) Concentration and Superoxide Dismutase (SOD) Activity in Lung Homogenates

Superoxide Dismutase (SOD) activity was measured following the manufacturer’s protocols (Cayman Chemicals, Ann Arbor, MI, USA). NADPH-dependent membrane lipid peroxidation was assessed as TBARS, using malondialdehyde as the standard (Sigma-Aldrich Fine Chemicals, St. Louis, MO, USA).

### 4.8. Isolation of Lung Mitochondria and Assessment of Mitochondrial Electron Transport Complexes

Mitochondria were extracted from mouse lungs using differential centrifugation following established protocols [64]. Briefly, a Dounce homogenizer was used to mince and blend the lungs with a mitochondrial isolation buffer containing 10 mM Tris base, 1 mM EDTA, and 0.32 M sucrose. After that, the homogenate was centrifuged for 10 min at 4 °C and 1000 rpm. After collecting the supernatant, it was centrifuged once again for 15 min at 4 °C and 15,000 rpm. For subsequent analysis, the resultant mitochondrial pellets were resuspended in isolation buffer and kept at temperatures lower than −80 °C. Using established techniques, the activities of complexes I, II, III, and IV were evaluated [64].

### 4.9. Quantification of Mitogen-Activated Protein Kinases (MAPKs) (Phospho-JNK/JNK, Phospho-ERK/ERK and Phospho-p38/P38) Ratios Expression in Lung Homogenates

Protein expressions for p-JNK/JNK, p-ERK/ERK, and p-p38/p38 ratios were quantified using the Western blot technique as described previously [76]. Lung tissues obtained from the mice were promptly snap-frozen in liquid nitrogen and preserved at −80 °C. Subsequently, the tissues were weighed, washed with saline, and homogenized in lysis buffer with a pH of 7.4. The homogenates were centrifuged for 20 min at 4 °C. The supernatants obtained were then utilized for protein quantification through the Pierce bicinchoninic acid protein assay kit (Thermo Scientific, Waltham, MA, USA). A protein sample of 35 µg was separated using 10% sodium dodecyl sulfate polyacrylamide gel electrophoresis and subsequently transferred to polyvinylidene difluoride membranes.

The membranes were blocked with 5% non-fat milk for 1 h at room temperature. They were then incubated overnight at 4 °C with rabbit monoclonal antibodies against p-ERK (Lot N° SC-7383, 1:2000, Santa Cruz, TX, USA), p-JNK (Lot N° SC-6254, 1:2000, Santa Cruz, TX, USA), p-p38 (Lot N° SC-7973, 1:2000, Santa Cruz, TX, USA), ERK (Lot N° SC-514302, 1:1000, Santa Cruz, TX, USA), JNK (Lot N° SC-7345, 1:1000, Santa Cruz, TX, USA), and p38 (Lot N° SC-7972, 1:1000, Santa Cruz, TX, USA). After washing the membranes 3 times for 10 min each, they were incubated for 2 h at room temperature with a goat anti-rabbit IgG horseradish peroxidase-conjugated secondary antibody (Lot N° ab6728, Abcam, Hong Kong, China).

The blots were then developed using the Pierce enhanced chemiluminescent plus Western blotting substrate kit (Thermo Scientific, Waltham, MA, USA). A chemical reaction occurs that emits light at 425 nm. Protein band densitometry was performed using an Azure biosystem (Azure sapphire biomolecular imager, Dublin, CA, USA). Finally, the blots were reprobed with a mouse monoclonal β-actin antibody (Lot N° SC-47778, 1:10,000, Santa Cruz, TX, USA) to serve as a loading control.

### 4.10. Statistical Analysis

All statistical analyses and graphs were generated using GraphPad Prism Version 7 for Windows software (GraphPad Software Inc., San Diego, CA, USA). To assess whether the measured parameters were normally distributed, the Kolmogorov-Smirnov test was first used. Normally distributed data were tested using one-way analysis of variance (ANOVA), followed by Holm–Sidak’s multiple comparisons test. Data that were not normally distributed (plasma cells, neutrophil polymorphs, focal interstitial fibrosis, CXCL1, and LDH) in the lung (numbers of lymphocytes and neutrophil polymorphs, CXCL1, CXCL2, CCL2, IL1β, IL-6, and TNFα) in BALF and (CXCL2, IL1β, IL-6, and TNFα) in plasma were tested using Kruskal-Wallis test followed by Dunn’s multiple comparison test. Thus, we have taken into consideration the comparisons of air vs. O-WPS, air vs. R-WPS, and O-WPS vs. R-WPS for all the assessed parameters. The data are presented as mean ± SEM (standard error of the mean).

## Data Availability

The data that support the findings of this study are available from the corresponding author, Abderrahim Nemmar, upon reasonable request.

## References

[1] Almomen S., Aldossari M., Khaleel Y., Altamimi M., Alharbi O., Alsuwaydani A., Almutairi M., Alyousef S., Hafiz R., Alshomer F. (2023). Effect of glycerol concentration on levels of toxicants emissions from water-pipe tobacco smoking (WTS). BMC Public Health.

[2] Chaouachi K. (2006). A critique of the WHO TobReg’s “Advisory Note” report entitled: “Waterpipe tobacco smoking: Health effects, research needs and recommended actions by regulators”. J. Negat. Results Biomed..

[3] Martinasek M.P., McDermott R.J., Martini L. (2011). Waterpipe (hookah) tobacco smoking among youth. Curr. Probl. Pediatr. Adolesc. Health Care.

[4] Jukema J.B., Bagnasco D.E., Jukema R.A. (2014). Waterpipe smoking: Not necessarily less hazardous than cigarette smoking: Possible consequences for (cardiovascular) disease. Neth. Heart J. Mon. J. Neth. Soc. Cardiol. Neth. Heart Found..

[5] Aljarrah K., Ababneh Z.Q., Al-Delaimy W.K. (2009). Perceptions of hookah smoking harmfulness: Predictors and characteristics among current hookah users. Tob. Induc. Dis..

[6] Maziak W., Taleb Z.B., Bahelah R., Islam F., Jaber R., Auf R., Salloum R.G. (2015). The global epidemiology of waterpipe smoking. Tob. Control.

[7] Zheng Z., Xie Z., Li D. (2022). Discussion of waterpipe tobacco smoking on reddit. Heliyon.

[8] Rashidian H., Hadji M., Ansari-Moghaddam A., Bakhshi M., Nejatizadeh A., Marzban M., Rezaianzadeh A., Seyyedsalehi M.S., Moradi A., Gholipour M. (2024). Association between waterpipe smoking and lung cancer: A multicentre case-control study in Iran. Public Health.

[9] Jawad M., Bakir A., Ali M., Grant A. (2015). Impact of Waterpipe Tobacco Pack Health Warnings on Waterpipe Smoking Attitudes: A Qualitative Analysis among Regular Users in London. BioMed Res. Int..

[10] Bhatnagar A., Maziak W., Eissenberg T., Ward K.D., Thurston G., King B.A., Sutfin E.L., Cobb C.O., Griffiths M., Goldstein L.B. (2019). Water Pipe (Hookah) Smoking and Cardiovascular Disease Risk: A Scientific Statement From the American Heart Association. Circulation.

[11] Jacob P., Abu Raddaha A.H., Dempsey D., Havel C., Peng M., Yu L., Benowitz N.L. (2013). Comparison of nicotine and carcinogen exposure with water pipe and cigarette smoking. Cancer Epidemiol. Biomark. Prev..

[12] Blank M.D., Cobb C.O., Kilgalen B., Austin J., Weaver M.F., Shihadeh A., Eissenberg T. (2011). Acute effects of waterpipe tobacco smoking: A double-blind, placebo-control study. Drug Alcohol Depend..

[13] Khabour O.F., Alzoubi K.H., Bani-Ahmad M., Dodin A., Eissenberg T., Shihadeh A. (2012). Acute exposure to waterpipe tobacco smoke induces changes in the oxidative and inflammatory markers in mouse lung. Inhal. Toxicol..

[14] Wong J., Magun B.E., Wood L.J. (2016). Lung inflammation caused by inhaled toxicants: A review. Int. J. Chronic Obstr. Pulm. Dis..

[15] Salameh P., Waked M., Khoury F., Akiki Z., Nasser Z., Abou Abbass L., Dramaix M. (2012). Waterpipe smoking and dependence are associated with chronic bronchitis: A case-control study in Lebanon. East. Mediterr. Health J..

[16] She J., Yang P., Wang Y., Qin X., Fan J., Wang Y., Gao G., Luo G., Ma K., Li B. (2014). Chinese water-pipe smoking and the risk of COPD. Chest.

[17] Nemmar A., Raza H., Yuvaraju P., Beegam S., John A., Yasin J., Hameed R.S., Adeghate E., Ali B.H. (2013). Nose-only water-pipe smoking effects on airway resistance, inflammation, and oxidative stress in mice. J. Appl. Physiol..

[18] Nemmar A., Al-Salam S., Yuvaraju P., Beegam S., Yasin J., Ali B.H. (2016). Chronic Exposure to Water-Pipe Smoke Induces Alveolar Enlargement, DNA Damage and Impairment of Lung Function. Cell. Physiol. Biochem. Int. J. Exp. Cell. Physiol. Biochem. Pharmacol..

[19] Inoue-Choi M., Christensen C.H., Rostron B.L., Cosgrove C.M., Reyes-Guzman C., Apelberg B., Freedman N.D. (2020). Dose-Response Association of Low-Intensity and Nondaily Smoking With Mortality in the United States. JAMA Netw. Open.

[20] Hamadi N., Beegam S., Zaaba N.E., Elzaki O., Ali B.H., Nemmar A. (2022). Comparative Study on the Chronic Vascular Responses Induced by Regular Versus Occasional Waterpipe Smoke Inhalation in Mice. Cell. Physiol. Biochem. Int. J. Exp. Cell. Physiol. Biochem. Pharmacol..

[21] Hamadi N., Al-Salam S., Beegam S., Zaaba N.E., Elzaki O., Nemmar A. (2024). Impact of prolonged exposure to occasional and regular waterpipe smoke on cardiac injury, oxidative stress and mitochondrial dysfunction in male mice. Front. Physiol..

[22] Nemmar A., Beegam S., Zaaba N.E., Elzaki O., Pathan A., Ali B.H. (2023). Waterpipe smoke inhalation induces lung injury and aortic endothelial dysfunction in mice. Physiol. Res..

[23] Nakhaee M.R., Zolfaghari M.R., Joukar S., Nakhaee N., Masoumi-Ardakani Y., Iranpour M., Nazari M. (2020). Swimming Exercise Training Attenuates the Lung Inflammatory Response and Injury Induced by Exposing to Waterpipe Tobacco Smoke. Addict. Health.

[24] Khabour O.F., Alzoubi K.H., Al-Sawalha N., Ahmad M.B., Shihadeh A., Eissenberg T. (2018). The effect of chronic exposure to waterpipe tobacco smoke on airway inflammation in mice. Life Sci..

[25] Nemmar A., Al-Salam S., Beegam S., Zaaba N.E., Ali B.H. (2021). Effect of smoking cessation on chronic waterpipe smoke inhalation-induced airway hyperresponsiveness, inflammation, and oxidative stress. Am. J. Physiology. Lung Cell. Mol. Physiol..

[26] Braber S., Henricks P.A., Nijkamp F.P., Kraneveld A.D., Folkerts G. (2010). Inflammatory changes in the airways of mice caused by cigarette smoke exposure are only partially reversed after smoking cessation. Respir. Res..

[27] Woodruff P.G., Ellwanger A., Solon M., Cambier C.J., Pinkerton K.E., Koth L.L. (2009). Alveolar macrophage recruitment and activation by chronic second hand smoke exposure in mice. Copd.

[28] Pouwels S.D., Zijlstra G.J., van der Toorn M., Hesse L., Gras R., Ten Hacken N.H., Krysko D.V., Vandenabeele P., de Vries M., van Oosterhout A.J. (2016). Cigarette smoke-induced necroptosis and DAMP release trigger neutrophilic airway inflammation in mice. Am. J. Physiol. Lung Cell. Mol. Physiol..

[29] Jia J., Conlon T.M., Ballester Lopez C., Seimetz M., Bednorz M., Zhou-Suckow Z., Weissmann N., Eickelberg O., Mall M.A., Yildirim A. (2016). Cigarette smoke causes acute airway disease and exacerbates chronic obstructive lung disease in neonatal mice. Am. J. Physiol. Lung Cell. Mol. Physiol..

[30] Moazed F., Burnham E.L., Vandivier R.W., O’Kane C.M., Shyamsundar M., Hamid U., Abbott J., Thickett D.R., Matthay M.A., McAuley D.F. (2016). Cigarette smokers have exaggerated alveolar barrier disruption in response to lipopolysaccharide inhalation. Thorax.

[31] Tanigawa T., Araki S., Nakata A., Kitamura F., Yasumoto M., Sakurai S., Kiuchi T. (1998). Increase in memory (CD4+CD29+ and CD4+CD45RO+) T and naive (CD4+CD45RA+) T-cell subpopulations in smokers. Arch. Environ. Health.

[32] Coussens L.M., Werb Z. (2002). Inflammation and cancer. Nature.

[33] Betsuyaku T., Hamamura I., Hata J., Takahashi H., Mitsuhashi H., Adair-Kirk T.L., Senior R.M., Nishimura M. (2008). Bronchiolar chemokine expression is different after single versus repeated cigarette smoke exposure. Respir. Res..

[34] Allais L., Verschuere S., Maes T., De Smet R., Devriese S., Gonzales G.B., Peeters H., Van Crombruggen K., Bachert C., De Vos M. (2020). Translational research into the effects of cigarette smoke on inflammatory mediators and epithelial TRPV1 in Crohn’s disease. PLoS ONE.

[35] Tiwari N., Marudamuthu A.S., Tsukasaki Y., Ikebe M., Fu J., Shetty S. (2016). p53- and PAI-1-mediated induction of C-X-C chemokines and CXCR2: Importance in pulmonary inflammation due to cigarette smoke exposure. Am. J. Physiol. Lung Cell. Mol. Physiol..

[36] Bratcher P.E., Weathington N.M., Nick H.J., Jackson P.L., Snelgrove R.J., Gaggar A. (2012). MMP-9 cleaves SP-D and abrogates its innate immune functions in vitro. PLoS ONE.

[37] Ishii T., Abboud R.T., Wallace A.M., English J.C., Coxson H.O., Finley R.J., Shumansky K., Paré P.D., Sandford A.J. (2014). Alveolar macrophage proteinase/antiproteinase expression in lung function and emphysema. Eur. Respir. J..

[38] Zhou L., Le Y., Tian J., Yang X., Jin R., Gai X., Sun Y. (2019). Cigarette smoke-induced RANKL expression enhances MMP-9 production by alveolar macrophages. Int. J. Chronic Obstr. Pulm. Dis..

[39] Haegens A., Vernooy J.H., Heeringa P., Mossman B.T., Wouters E.F. (2008). Myeloperoxidase modulates lung epithelial responses to pro-inflammatory agents. Eur. Respir. J..

[40] Khabour O.F., Alzoubi K.H., Abu Thiab T.M., Al-Husein B.A., Eissenberg T., Shihadeh A.L. (2015). Changes in the expression and protein level of matrix metalloproteinases after exposure to waterpipe tobacco smoke. Inhal. Toxicol..

[41] Nemmar A., Al-Salam S., Beegam S., Yuvaraju P., Ali B.H. (2020). Comparative Study on Pulmonary Toxicity in Mice Induced by Exposure to Unflavoured and Apple- and Strawberry-Flavoured Tobacco Waterpipe Smoke. Oxidative Med. Cell. Longev..

[42] Atkinson J.J., Lutey B.A., Suzuki Y., Toennies H.M., Kelley D.G., Kobayashi D.K., Ijem W.G., Deslee G., Moore C.H., Jacobs M.E. (2011). The role of matrix metalloproteinase-9 in cigarette smoke-induced emphysema. Am. J. Respir. Crit. Care Med..

[43] Kim M.D., Baumlin N., Dennis J.S., Yoshida M., Kis A., Aguiar C., Schmid A., Mendes E., Salathe M. (2021). Losartan reduces cigarette smoke-induced airway inflammation and mucus hypersecretion. ERJ Open Res..

[44] Yu D., Liu X., Zhang G., Ming Z., Wang T. (2018). Isoliquiritigenin Inhibits Cigarette Smoke-Induced COPD by Attenuating Inflammation and Oxidative Stress via the Regulation of the Nrf2 and NF-κB Signaling Pathways. Front. Pharmacol..

[45] Nath D., Shivasekar M., Vinodhini V.M. (2022). Smoking Induces the Circulating Levels of Matrix Metalloproteinase-9 and Its Association with Cardiovascular Risk in Young Smokers. Medeni. Med. J..

[46] Klein R., Nagy O., Tóthová C., Chovanová F. (2020). Clinical and Diagnostic Significance of Lactate Dehydrogenase and Its Isoenzymes in Animals. Vet. Med. Int..

[47] Nemmar A., Al Hemeiri A., Al Hammadi N., Yuvaraju P., Beegam S., Yasin J., Elwasila M., Ali B.H., Adeghate E. (2015). Early pulmonary events of nose-only water pipe (shisha) smoking exposure in mice. Physiol. Rep..

[48] Anbarasi K., Sabitha K.E., Devi C.S. (2005). Lactate dehydrogenase isoenzyme patterns upon chronic exposure to cigarette smoke: Protective effect of bacoside A. Environ. Toxicol. Pharmacol..

[49] Ranawat P., Kaur N., Koul A. (2023). Modulation of cigarette smoke induced alterations by aqueous Ocimum sanctum leaf extract in pulmonary tissue of rodents. Sci. Rep..

[50] Zhou L., Jian T., Wan Y., Huang R., Fang H., Wang Y., Liang C., Ding X., Chen J. (2023). Luteolin Alleviates Oxidative Stress in Chronic Obstructive Pulmonary Disease Induced by Cigarette Smoke via Modulation of the TRPV1 and CYP2A13/NRF2 Signaling Pathways. Int. J. Mol. Sci..

[51] Nemmar A., Al-Salam S., Yuvaraju P., Beegam S., Ali B.H. (2018). Exercise Training Mitigates Water Pipe Smoke Exposure-Induced Pulmonary Impairment via Inhibiting NF-κB and Activating Nrf2 Signalling Pathways. Oxidative Med. Cell. Longev..

[52] Belchamber K., Hall D.A., Hourani S.M. (2014). Smoking enhances the proinflammatory effects of nucleotides on cytokine release from human lung. PLoS ONE.

[53] Sundar I.K., Rahman I. (2016). Gene expression profiling of epigenetic chromatin modification enzymes and histone marks by cigarette smoke: Implications for COPD and lung cancer. Am. J. Physiol. Lung Cell. Mol. Physiol..

[54] Frangie C., Daher J. (2022). Role of myeloperoxidase in inflammation and atherosclerosis (Review). Biomed. Rep..

[55] Park H.Y., Man S.F., Tashkin D., Wise R.A., Connett J.E., Anthonisen N.A., Sin D.D. (2013). The relation of serum myeloperoxidase to disease progression and mortality in patients with chronic obstructive pulmonary disease (COPD). PLoS ONE.

[56] Agustí A., Hogg J.C. (2019). Update on the Pathogenesis of Chronic Obstructive Pulmonary Disease. N. Engl. J. Med..

[57] Barnes P.J. (2022). Oxidative Stress in Chronic Obstructive Pulmonary Disease. Antioxidants.

[58] Hakim F., Hellou E., Goldbart A., Katz R., Bentur Y., Bentur L. (2011). The acute effects of water-pipe smoking on the cardiorespiratory system. Chest.

[59] Khan N.A., Sundar I.K., Rahman I. (2018). Strain- and sex-dependent pulmonary toxicity of waterpipe smoke in mouse. Physiol. Rep..

[60] Badran M., Laher I. (2020). Waterpipe (shisha, hookah) smoking, oxidative stress and hidden disease potential. Redox Biol..

[61] Alsaad A.M., Al-Arifi M.N., Maayah Z.H., Attafi I.M., Alanazi F.E., Belali O.M., Alhoshani A., Asiri Y.A., Korashy H.M. (2019). Genotoxic impact of long-term cigarette and waterpipe smoking on DNA damage and oxidative stress in healthy subjects. Toxicol. Mech. Methods.

[62] Martins S.G., Zilhão R., Thorsteinsdóttir S., Carlos A.R. (2021). Linking Oxidative Stress and DNA Damage to Changes in the Expression of Extracellular Matrix Components. Front. Genet..

[63] Grillo R., Khemiss M., da Silva Y.S. (2023). Cytotoxic and Genotoxic Effects of Waterpipe on Oral Health Status: Systematic review and meta-analysis. Sultan Qaboos Univ. Med. J..

[64] Nemmar A., Al-Salam S., Beegam S., Zaaba N.E., Elzaki O., Ali B.H. (2023). Waterpipe smoke inhalation potentiates cardiac oxidative stress, inflammation, mitochondrial dysfunction, apoptosis and autophagy in experimental hypertension. Biomed. Pharmacother. Biomed. Pharmacother..

[65] Tan D., Goerlitz D.S., Dumitrescu R.G., Han D., Seillier-Moiseiwitsch F., Spernak S.M., Orden R.A., Chen J., Goldman R., Shields P.G. (2008). Associations between cigarette smoking and mitochondrial DNA abnormalities in buccal cells. Carcinogenesis.

[66] Ballweg K., Mutze K., Königshoff M., Eickelberg O., Meiners S. (2014). Cigarette smoke extract affects mitochondrial function in alveolar epithelial cells. Am. J. Physiology. Lung Cell. Mol. Physiol..

[67] Wang Z., White A., Wang X., Ko J., Choudhary G., Lange T., Rounds S., Lu Q. (2020). Mitochondrial Fission Mediated Cigarette Smoke-induced Pulmonary Endothelial Injury. Am. J. Respir. Cell Mol. Biol..

[68] Cowan K.J., Storey K.B. (2003). Mitogen-activated protein kinases: New signaling pathways functioning in cellular responses to environmental stress. J. Exp. Biol..

[69] Chuang S.M., Wang I.C., Yang J.L. (2000). Roles of JNK, p38 and ERK mitogen-activated protein kinases in the growth inhibition and apoptosis induced by cadmium. Carcinogenesis.

[70] Broom O.J., Widjaya B., Troelsen J., Olsen J., Nielsen O.H. (2009). Mitogen activated protein kinases: A role in inflammatory bowel disease?. Clin. Exp. Immunol..

[71] Liu Q., Xu W.G., Luo Y., Han F.F., Yao X.H., Yang T.Y., Zhang Y., Pi W.F., Guo X.J. (2011). Cigarette smoke-induced skeletal muscle atrophy is associated with up-regulation of USP-19 via p38 and ERK MAPKs. J. Cell. Biochem..

[72] Khan D., Zhou H., You J., Kaiser V.A., Khajuria R.K., Muhammad S. (2024). Tobacco smoke condensate-induced senescence in endothelial cells was ameliorated by colchicine treatment via suppression of NF-κB and MAPKs P38 and ERK pathways activation. Cell Commun. Signal. CCS.

[73] Restivo I., Attanzio A., Giardina I.C., Di Gaudio F., Tesoriere L., Allegra M. (2022). Cigarette Smoke Extract Induces p38 MAPK-Initiated, Fas-Mediated Eryptosis. Int. J. Mol. Sci..

[74] Nemmar A., Yuvaraju P., Beegam S., John A., Raza H., Ali B.H. (2013). Cardiovascular effects of nose-only water-pipe smoking exposure in mice. Am. J. Physiology. Heart Circ. Physiol..

[75] Nemmar A., Al-Salam S., Yuvaraju P., Beegam S., Yasin J., Ali B.H. (2017). Chronic exposure to water-pipe smoke induces cardiovascular dysfunction in mice. Am. J. Physiology. Heart Circ. Physiol..

[76] Beegam S., Al-Salam S., Zaaba N.E., Elzaki O., Ali B.H., Nemmar A. (2024). Effects of Waterpipe Smoke Exposure on Experimentally Induced Chronic Kidney Disease in Mice. Int. J. Mol. Sci..

[77] Toukan Y., Hakim F., Bentur Y., Aharon-Peretz J., Elemy A., Gur M., Hanna M., Fisher T., Scherb I., Bentur L. (2020). The Effect of a 30-Min Water-Pipe Smoking Session on Cognitive Measures and Cardio-Pulmonary Parameters. Nicotine Tob. Res. Off. J. Soc. Res. Nicotine Tob..

[78] Zu G.X., Sun K.Y., Liu X.J., Tang J.Q., Huang H.L., Han T. (2024). Banxia xiexin decoction prevents the development of gastric cancer. World J. Clin. Oncol..

[79] Houchen C.J., Ghanem S., Kaartinen V., Bumann E.E. (2024). TGF-β signaling in the cranial neural crest affects late-stage mandibular bone resorption and length. Front. Physiol..

[80] Zaaba N.E., Al-Salam S., Beegam S., Elzaki O., Yasin J., Nemmar A. (2023). Catalpol Attenuates Oxidative Stress and Inflammation via Mechanisms Involving Sirtuin-1 Activation and NF-κB Inhibition in Experimentally-Induced Chronic Kidney Disease. Nutrients.

[81] Hardy R.D., Coalson J.J., Peters J., Chaparro A., Techasaensiri C., Cantwell A.M., Kannan T.R., Baseman J.B., Dube P.H. (2009). Analysis of pulmonary inflammation and function in the mouse and baboon after exposure to Mycoplasma pneumoniae CARDS toxin. PLoS ONE.

[82] Bubeck S.S., Cantwell A.M., Dube P.H. (2007). Delayed inflammatory response to primary pneumonic plague occurs in both outbred and inbred mice. Infect. Immun..

[83] Hartmann A., Speit G. (1997). The contribution of cytotoxicity to DNA-effects in the single cell gel test (comet assay). Toxicol. Lett..

[84] Hartmann A., Speit G. (1995). Genotoxic effects of chemicals in the single cell gel (SCG) test with human blood cells in relation to the induction of sister-chromatid exchanges (SCE). Mutat. Res..

